# Zona pellucida-inspired smart viral vectors: a biomimetic platform for multi-modal immune evasion and triple-stimuli responsive gene delivery

**DOI:** 10.3389/fbioe.2026.1949512

**Published:** 2026-09-29

**Authors:** Ziyi Xu, Xiaobing Luo, Yueming Li, Junrong Qian, Guangming Xu

**Affiliations:** 1 Xi’an Technological University Affiliated High School, Xi’an, Shaanxi, China; 2 Independent Researcher in Molecular Biology, Xi’an, Shaanxi, China; 3 Xi’an No.3 High School, Xi’an, Shaanxi, China; 4 School of Mechatronic Engineering, Xi’an Technological University, Xi’an, Shaanxi, China

**Keywords:** biomimetic gene delivery, controlled release, immune evasion, smart viral vectors, triple-stimuli responsive, zona pellucida

## Abstract

Pre-existing immunity and off-target toxicity limit the clinical translation of adeno-associated virus (AAV) vectors. Herein, we present a Zona Pellucida-inspired Smart Viral Vector (ZP-SVV) platform as a biomimetic design hypothesis that repurposes the evolutionarily optimized architecture of ZP glycoproteins to engineer intelligent viral vector surfaces. The system integrates a self-assembling ZP glycoprotein coating—covalently crosslinked filamentous meshes formed via ZP-N/ZP-C domain polymerization, yielding a tunable glycan shield with defined inter-filament pores—with a triple-stimuli responsive architecture of protease-sensitive linkers, pH-gated histidine-rich domains, and redox-sensitive disulfide bonds that releases payload only after sequential triggering, and with multi-modal immune evasion that pairs dense glycan shielding with extracellular matrix mimicry to evade humoral and cellular immune surveillance while preserving receptor accessibility. This biomimetic design is intended to enhance safety and efficiency relative to conventional PEGylation or membrane-wrapping strategies, with theoretical modeling predicting potentially improved tumor accumulation and controlled intracellular release. Presented as a testable design framework rather than a validated therapy, the ZP-SVV platform offers a new paradigm for rational vector engineering in reproductive medicine and oncolytic virotherapy.

## 1 Introduction

### 1.1 Current landscape and limitations of viral gene delivery

Viral gene delivery, particularly via adeno-associated virus (AAV) vectors, has demonstrated transformative therapeutic potential across monogenic disorders, hemophilia, inherited retinal diseases, and increasingly, complex indications such as cancer and cardiovascular disorders (Wang D et al., 2019). The clinical approvals of voretigene neparvovec for RPE65-mediated retinal dystrophy, onasemnogene abeparvovec for spinal muscular atrophy, and etranacogene dezaparvovec for hemophilia B have established AAV as the leading platform for *in vivo* gene transfer (High and Roncarolo, 2019; Mendell et al., 2021). With over 200 ongoing clinical trials (Mendell et al., 2021) and a rapidly expanding market projected to reach multi-billion USD valuation within the next decade, AAV-based therapies are poised for broad clinical adoption. Nevertheless, several interconnected biological barriers constrain their widespread translation.

Pre-existing immunity against common AAV serotypes constitutes the most formidable obstacle. Epidemiological studies across diverse populations demonstrate that 30%–60% of humans harbor neutralizing antibodies (NAbs) against prevalent serotypes (AAV2, AAV5, AAV8, AAV9) (Calcedo et al., 2009; Ginn et al., 2024; Liu et al., 2014). These NAbs, arising from natural exposure to wild-type AAV or related parvoviruses during childhood, neutralize vector infectivity through multiple mechanisms: steric blockade of receptor attachment, prevention of endosomal entry, and Fc receptor-mediated clearance of immune complexes by Kupffer cells and splenic macrophages (Ravindra Kumar et al., 2020; Schulz et al., 2023). Consequently, NAb-positive patients are frequently excluded from clinical trials, substantially restricting the addressable patient population (Finkel et al., 2017).

Beyond humoral immunity, cellular immune responses directed against AAV capsids present additional hurdles. The landmark hemophilia B trial employing AAV8-mediated factor IX gene transfer revealed CD8^+^ T-cell-mediated elimination of transduced hepatocytes, resulting in loss of transgene expression and necessitating immunosuppressive regimens (Nathwani et al., 2014). High vector doses required for systemic delivery to large organs further exacerbate immunogenicity by increasing antigenic load, elevating risks of cytotoxic T lymphocyte (CTL) activation and inflammatory responses (Perrin et al., 2019). Recent high-dose trials have additionally identified complement system activation as an emerging safety concern, with thrombotic microangiopathy and hepatotoxicity observed at doses exceeding 3 × 10^13^ vector genomes per kilogram (Hinderer et al., 2018; Chand et al., 2021).

Equally critical are limitations in targeting precision and controlled intracellular release. Current AAV vectors rely primarily on passive targeting governed by natural capsid tropism. Although directed evolution has generated variants with altered tropism, achieving selective targeting of diseased tissues while sparing healthy tissues remains elusive (Deverman et al., 2018). Following receptor-mediated endocytosis, AAV particles traffic through the endosomal system where they must escape prior to lysosomal degradation (Domenge et al., 2019). The natural endosomal escape mechanism involving the VP1 phospholipase A2 domain is inefficient, with a substantial fraction of internalized vectors failing to reach the cytosol. This limitation is particularly acute for applications requiring precise spatiotemporal control—such as germline gene correction, where premature expression could disrupt embryonic development, or tumor-specific oncolytic activation, where off-target expression risks systemic toxicity.

### 1.2 Biomimetic strategies in vector engineering

The limitations of conventional AAV engineering have motivated exploration of biomimetic strategies leveraging nature’s solutions to analogous biological challenges. PEGylation, the covalent attachment of polyethylene glycol (PEG) chains to the capsid surface, has been the predominant stealthing strategy. PEGylation creates a hydrophilic layer that sterically excludes antibodies and complement components, reducing immune recognition and extending circulation time (Fu et al., 2025). However, PEGylation introduces significant trade-offs: flexible PEG chains mask capsid receptor-binding sites, reducing cellular uptake and transduction efficiency. Furthermore, PEGylation does not provide active targeting capabilities, and the PEG layer itself can elicit anti-PEG antibodies in some patients, compromising efficacy upon repeated administration (Simberg et al., 2025). The non-degradable nature of PEG means the stealth layer persists indefinitely, preventing receptor-mediated interactions even after reaching target tissue.

Cell membrane coating represents an advanced biomimetic strategy gaining substantial attention. By extracting membranes from red blood cells, platelets, cancer cells, or other cell types and fusing them around viral cores, researchers have created vectors displaying natural self-markers (Fang et al., 2023). Red blood cell membrane (RBCM) wrapping demonstrates remarkable immune evasion through CD47 display, a phagocytosis-inhibitory signal engaging signal regulatory protein-α (SIRP-α) on macrophages. Complete membrane coating physically masks AAV capsid epitopes from antibody recognition. However, RBCM coating introduces substantial manufacturing complexity associated with membrane extraction, purification, and fusion procedures. Resulting vectors exhibit batch-to-batch variability in membrane composition, and complete surface coverage interferes with vector–cell interactions by masking capsid receptor-binding sites (Krishnan et al., 2024). The membrane coating also lacks stimuli-responsive properties, preventing selective uncoating in response to disease-specific cues.

Exosome-based delivery represents another biomimetic approach leveraging natural extracellular vesicles. Exosomes are endogenous nanovesicles mediating intercellular communication with inherent biocompatibility, low immunogenicity, and natural targeting properties (Pegtel and Gould, 2019). However, exosomes face significant challenges including limited loading capacity for large nucleic acids, heterogeneity in size and surface marker composition, and difficulties in scalable manufacturing. These existing strategies reveal a fundamental tension: achieving immune evasion often comes at the cost of reduced targeting and controlled release capabilities. There is a clear need for a unified biomimetic platform simultaneously addressing immune evasion, active targeting, and controlled intracellular release without associated trade-offs (Mitchell et al., 2021).

### 1.3 Zona pellucida architecture as a biomimetic blueprint

The mammalian zona pellucida (ZP) represents one of the most evolutionarily refined extracellular matrices, functioning as a critical interface between the oocyte and its environment across approximately 300 million years of metazoan reproduction (Litscher and Wassarman, 2007). This specialized extracellular matrix performs multiple simultaneous functions directly analogous to requirements of advanced gene delivery vectors: protecting the female gamete from environmental insults and pathogens, permitting selective molecular exchange across the matrix, mediating species-specific sperm recognition through carbohydrate-mediated interactions, and preventing polyspermy through post-fertilization hardening mechanisms that render the matrix impermeable (Wassarman and Litscher, 2018; Boja et al., 2003). The architectural complexity enabling this multifunctionality provides compelling rationale for biomimetic adaptation.

In humans, the ZP is composed of four distinct glycoproteins (ZP1, ZP2, ZP3, and ZP4), each contributing unique structural properties. The structural foundation centers on the ZP domain (ZPD), an approximately 260-amino-acid module characterized by eight conserved cysteine residues forming intramolecular disulfide bonds and high β-strand content adopting an immunoglobulin-like fold, conferring exceptional mechanical stability (Monné and Jovine, 2011). The ZPD exhibits bipartite organization with ZP-N and ZP-C subdomains separated by a protease-sensitive linker region, essential for filament polymerization and matrix assembly (Han et al., 2010). ZP1 and ZP4 possess additional trefoil domains absent in ZP2 and ZP3, contributing to inter-filament cross-linking. The hierarchical assembly follows precise stoichiometry: ZP3 provides the filament scaffold through head-to-tail polymerization, ZP1 cross-links filaments via intermolecular disulfide bonds, and ZP2 oligomerizes after fertilization to harden the matrix (Jovine et al., 2002; Wassarman, 2008).

The ZP matrix exhibits properties directly translatable to gene delivery vector design. First, the self-assembling nature enables formation of stable, covalently crosslinked matrices without complex chemical conjugation. Polymerization is essentially irreversible under physiological conditions due to extensive buried surface area at the ZP-C/ZP-N interface (Raj et al., 2017). Second, dense glycosylation creates a glycan shield masking underlying protein epitopes, conceptually analogous to the glycan shield employed by HIV-1 to evade neutralizing antibodies (Li et al., 2023). Third, the filamentous architecture creates a porous mesh with defined inter-filament spacing that maintains selective permeability while excluding large immune complexes. Fourth, the ZP undergoes controlled remodeling through protease-mediated cleavage, demonstrating stimuli-responsive behavior optimized over evolutionary timescales (Inoue et al., 2005).

### 1.4 Design philosophy and innovation strategy

The zona pellucida-inspired smart viral vector (ZP-SVV) platform was conceptualized by systematically repurposing ZP glycoprotein architecture to engineer intelligent viral vector surfaces. The design integrates three core innovations collectively addressing limitations of current approaches (Yoo et al., 2011). The first innovation involves self-assembling ZP glycoprotein coatings forming covalently crosslinked filamentous meshes around AAV capsids through genetically encoded polymerization. Unlike synthetic coatings requiring chemical conjugation, the ZP coating assembles spontaneously through domain-mediated interactions, achieving uniform coverage without complex manufacturing. The covalent crosslinks provide mechanical stability superior to non-covalent coatings, while filamentous architecture preserves receptor accessibility through inter-filament pores (Gupta et al., 2009).

Multi-modal immune evasion forms the second layer of the design. Dense glycan shielding is combined with extracellular matrix (ECM) mimicry, and rather than relying on any single mechanism the platform stacks complementary strategies: glycan-mediated steric exclusion, sialic acid-mediated complement regulation, siglec-mediated phagocytosis inhibition, and ECM mimicry presenting familiar molecular patterns (Phelps et al., 2012; Li et al., 2015). Smart responsive elements complete the design. Unlike conventional single-stimulus systems, the platform requires sequential progression through three triggers—tumor-associated proteases for initial remodeling, endosomal acidification for membrane escape, and cytosolic glutathione for final disassembly—creating a molecular AND-gate that minimizes premature release (Mura et al., 2013). AAV serotypes 1 and DJ were selected as viral cores based on their demonstrated ZP-penetrating capability and established manufacturing infrastructure (Zincarelli et al., 2008; Romeo et al., 2020) (Figure 1).

**FIGURE 1:**
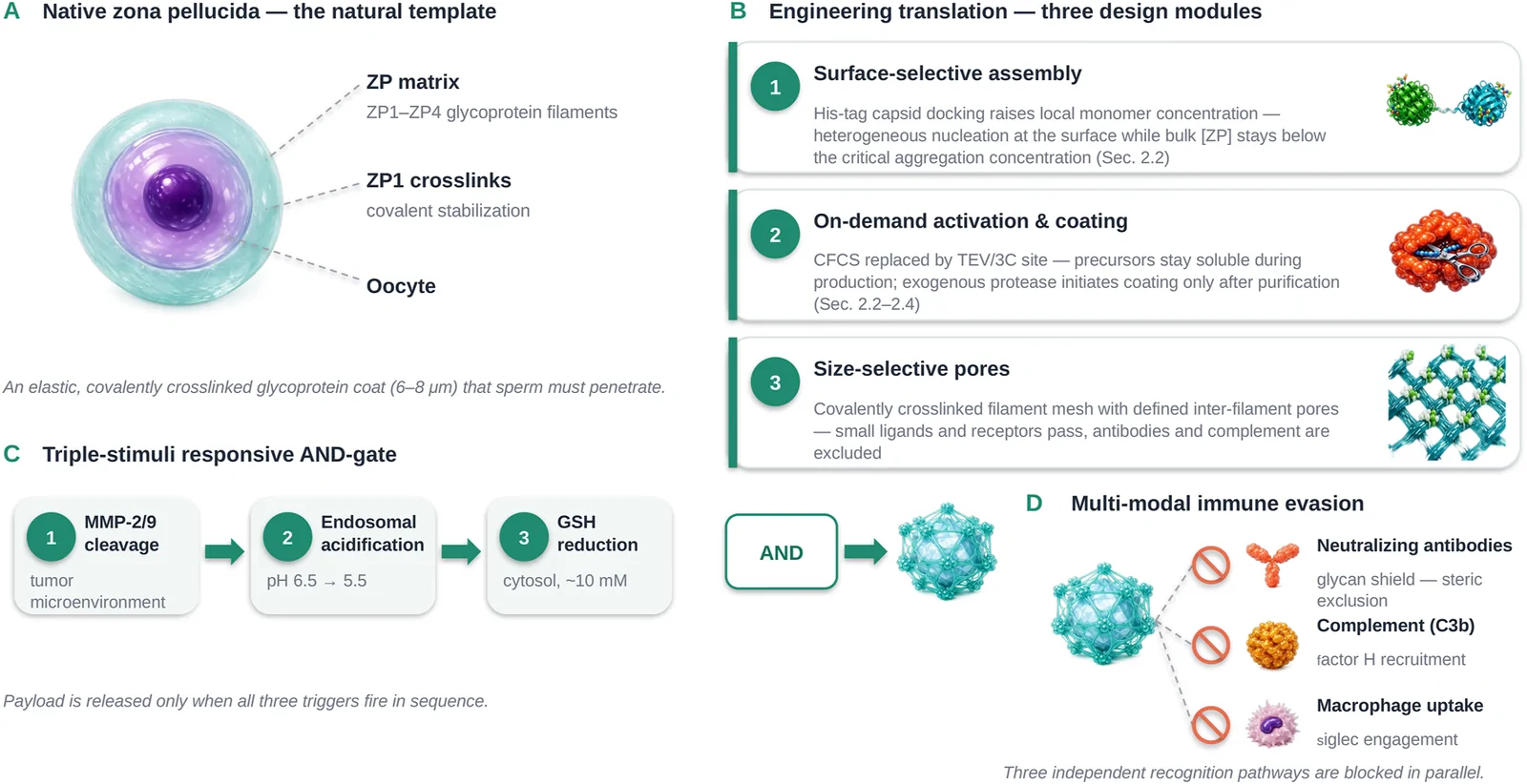
Design of the ZP-SVV biomimetic platform. **(A)** Architecture of the native zona pellucida: ZP1–ZP4 glycoproteins polymerize via ZP-N/ZP-C domains into a covalently crosslinked filamentous matrix surrounding the oocyte. **(B)** Engineering strategy: soluble, polymerization-incompetent ZP glycoproteins are activated on demand by exogenous protease and self-assemble on adeno-associated virus (AAV) capsids through affinity tag-mediated nucleation, forming a filamentous mesh with defined inter-filament pores. **(C)** Triple-stimuli responsive AND-gate logic: sequential triggering by tumor-associated proteases (MMP-2/9), endosomal acidification, and cytosolic glutathione (GSH). **(D)** Multi-modal immune evasion mechanisms: glycan shielding against neutralizing antibodies (NAbs), complement regulation via factor H recruitment, and phagocytosis inhibition via siglec engagement.

## 2 ZP-derived self-assembling coating

### 2.1 ZP domain polymerization mechanism

The self-assembly capability of ZP glycoproteins represents a compelling feature for biomimetic vector design, offering a genetically encoded mechanism for stable surface coating without complex chemical conjugation. In native oocytes, polymerization follows a precisely defined sequence elucidated through decades of reproductive biology research. Precursor proteins are synthesized with the ZP domain and an appended C-terminal propeptide (CTP) containing the external hydrophobic patch (EHP) (Kiefer and Saling, 2002). The EHP prevents premature polymerization by sterically masking the ZP-C polymerization interface, ensuring polymerization occurs only at appropriate times and locations. At the trans-Golgi network, furin-like proprotein convertase recognizes the consensus furin cleavage site within the CTP and cleaves it, exposing the ZP-C interface and initiating head-to-tail filament assembly through ZP-C/ZP-N intermolecular interactions (Litscher and Wassarman, 2020).

Structural studies of ZP-domain proteins have revealed molecular details of ZP domain polymerization. The ZP-N subdomain adopts an immunoglobulin-like β-sandwich fold, while the ZP-C subdomain forms a distinct globular structure. The polymerization interface involves a complex network of hydrogen bonds, hydrophobic contacts, and electrostatic interactions across an extensive buried surface area of approximately 1,500–2,000 Å^2^ per interface, driving essentially irreversible assembly under physiological conditions (Bokhove et al., 2016; Schaeffer et al., 2009). This extensive buried interface explains the remarkable stability of ZP filaments, which withstand mechanical stress while retaining limited flexibility necessary for matrix function.

### 2.2 Engineering strategies for controlled capsid coating

Three complementary engineering strategies were designed to adapt natural ZP polymerization for controlled vector coating while preserving essential architectural features.

Strategy I: Exogenous Protease-Controlled Activation. The native consensus furin cleavage site (CFCS), characterized by an arginine-X-lysine/arginine-arginine motif, is recognized by endogenous furin expressed in Chinese hamster ovary (CHO) and HEK293 cells used for AAV manufacturing (Thomas, 2002). To prevent premature polymerization during vector production, the CFCS was modified to sequences resistant to endogenous furin yet specifically recognized by exogenous proteases such as tobacco, etch virus (TEV) protease or human rhinovirus 3C protease. This modification enables a controlled activation paradigm: ZP proteins are expressed and purified in a soluble, non-polymerized state, with polymerization initiated exclusively upon addition of exogenous protease in the presence of AAV capsids. Such spatiotemporal control ensures coating formation occurs specifically at capsid surfaces rather than generating soluble aggregates (Hoodbhoy and Dean, 2004).

Strategy II: Interface Mutagenesis for Kinetic Tuning. The ZP-C/ZP-N interaction involves hydrogen bonds, hydrophobic contacts, and electrostatic interactions across an extensive interface. Alanine-scanning mutagenesis and computational modeling can modulate binding affinity and polymerization rate (Cunningham and Wells, 1989). Mutations weakening interface interactions generate variants with slower kinetics, producing thinner, more permeable coatings suited for applications requiring rapid payload release. Conversely, strengthening mutations yield thicker coatings providing enhanced immune protection where prolonged circulation is desired.

Strategy III: ZP Domain as Fusion Tag for Co-polymerization. By fusing targeting ligands, pH-responsive peptides, or glycosylation motifs to the ZP domain, functional elements incorporate into growing filaments during polymerization (Jovine et al., 2005). This co-polymerization approach achieves uniform molecular-level distribution throughout the coating rather than surface-only display, enabling integration of structural, targeting, and responsive functions without the steric interference typical of *post hoc* chemical conjugation.

Selective capsid-surface assembly versus bulk polymerization. The coating process must drive ZP polymerization at the capsid surface while suppressing uncontrolled aggregation in solution. Capsid-displayed affinity tags recruit ZP monomers and raise their local concentration, so assembly initiates by heterogeneous, surface-catalyzed nucleation whose free-energy barrier sits well below that of homogeneous nucleation; even a modest barrier reduction translates into orders-of-magnitude differences in nucleation rate. During activation, the bulk monomer concentration is held below the critical aggregation concentration, making spontaneous filament formation in solution thermodynamically disfavored within the coating window. Chain-terminating truncation mutants then quench the reaction at the target thickness, with dynamic light scattering and quartz crystal microbalance monitoring serving as real-time gates. Because the precursor proteins retain their C-terminal propeptide and external hydrophobic patch until exogenous protease activation, they remain soluble through production and purification and cannot self-associate prematurely. The quantitative rationale behind these choices is developed in Sections 2.4 and 2.5.

### 2.3 Self-assembly kinetics and quality control

The kinetics of ZP domain polymerization on capsid surfaces can be theoretically modeled using principles from nucleation-dependent amyloid assembly. The polymerization follows a sigmoidal progression comprising a lag phase, a growth phase, and a plateau phase. During the lag phase, ZP proteins undergo conformational rearrangement at the capsid surface, forming critical nuclei for filament elongation. The duration of this phase depends on ZP protein concentration, solution conditions, and the presence of affinity tags on the capsid surface that serve as nucleation sites (Jovine et al., 2002). At optimal concentrations of 10–50 μM ZP protein, the lag phase typically spans 5–15 min, followed by rapid growth over 30–60 min until reaching plateau (Kiefer and Saling, 2002).

Quality control of the self-assembled coating presents unique challenges distinct from conventional chemical conjugation. Process analytical technology for real-time monitoring includes dynamic light scattering to assess coating uniformity, transmission electron microscopy for filament morphology, and quartz crystal microbalance with dissipation monitoring for thickness determination. The self-assembling nature simplifies manufacturing compared to chemical methods, as the coating forms spontaneously upon mixing under controlled conditions, potentially reducing batch-to-batch variability. However, glycosylation heterogeneity remains a significant challenge requiring glycoengineered cell lines producing homogeneous glycoforms and *in vitro* glycan remodeling using glycosyltransferases for defined structures (Meuris et al., 2014).

Producing the required glycoforms at scale. The multi-modal immune evasion functions depend on highly sialylated, complex-type N-glycans, which the standard AAV manufacturing hosts do not produce efficiently: HEK293 cells generate complex N-glycans but with limited terminal α2,6-sialylation, and CHO cells predominantly yield α2,3-linked sialoforms owing to insufficient ST6GAL1 activity. Three complementary solutions are available—host-cell glycoengineering (ST6GAL1 overexpression to raise α2,6-sialylation (Bragonzi et al., 2000), or GlycoDelete-type streamlining of Golgi processing to yield homogeneous sialylated glycoforms (Meuris et al., 2014)); chemoenzymatic *in vitro* remodeling of purified, coated vectors with galactosyltransferase and α2,6-sialyltransferase to install defined terminal structures after assembly; and glycoform-directed quality control using lectin-affinity profiling and mass spectrometric glycan mapping as release assays. We regard glycoform homogeneity as one of the principal feasibility risks of the platform and include it explicitly in the validation agenda (Section 5.5).

### 2.4 Mesh architecture and selective permeability

Controlled self-assembly on capsid surfaces requires preventing uncontrolled aggregation while achieving uniform coatings of defined thickness. Affinity tag-mediated nucleation utilizes capsid-displayed peptides such as polyhistidine tags to recruit ZP proteins specifically to vector surfaces, ensuring polymerization initiates at capsid surfaces rather than in bulk solution (Girod et al., 1999). Concentration and condition optimization at pH 7.0–7.4, physiological ionic strength, and 37 °C modulates polymerization kinetics to favor controlled growth. Truncated ZP proteins lacking polymerization-competent interfaces serve as chain terminators, limiting filament extension and controlling coating thickness.

Controlling assembly stoichiometry and preventing empty “ghost” filaments. The vector-to-ZP stoichiometry is constrained by capsid geometry: an AAV capsid (diameter ≈ 25 nm) presents ≈ 2,000 nm^2^ of surface area, and with an estimated filament footprint of ≈ 25–30 nm^2^ per incorporated ZP monomer, a complete two-to-three-layer coat requires on the order of 200–500 monomers per capsid (a design target to be confirmed by cryo-EM). Coating reactions are accordingly performed at a defined capsid:ZP molar ratio (≈1:300–1:500), with capsids present during protease activation so that nucleation occurs on capsid surfaces, and with ZP monomer held sub-stoichiometric relative to the available nucleation sites to preclude a free-monomer reservoir capable of bulk nucleation. A two-stage protocol further suppresses the formation of empty “ghost” filaments: a nucleation stage at low activated-monomer concentration is followed by an elongation stage in which additional monomer is fed onto existing capsid-bound filaments; residual activated monomers are then quenched with polymerization-incompetent chain terminators, and any filamentous material not associated with capsids is removed by size-exclusion or ion-exchange polishing. Bulk filament formation is monitored by negative-stain electron microscopy and analytical ultracentrifugation as in-process controls.

The resulting ZP coating exhibits mesh-like architecture with inter-filament spacing sufficient to permit diffusion of small molecules and maintenance of receptor accessibility while restricting access of large immune complexes. Individual filaments, estimated at 5–10 nm in diameter from native ZP structural studies (a design target to be confirmed by cryo-EM), create porous networks preserving AAV capsid receptor engagement while sterically blocking antibodies and complement components (Avella et al., 2014). This selective permeability fundamentally differs from PEGylation, where dense PEG layers block all macromolecular interactions regardless of size or function. The ZP mesh provides size-selective and charge-selective barriers discriminating between functional receptor interactions and pathological immune recognition (Weinbaum et al., 2007) (Figure 2).

**FIGURE 2:**
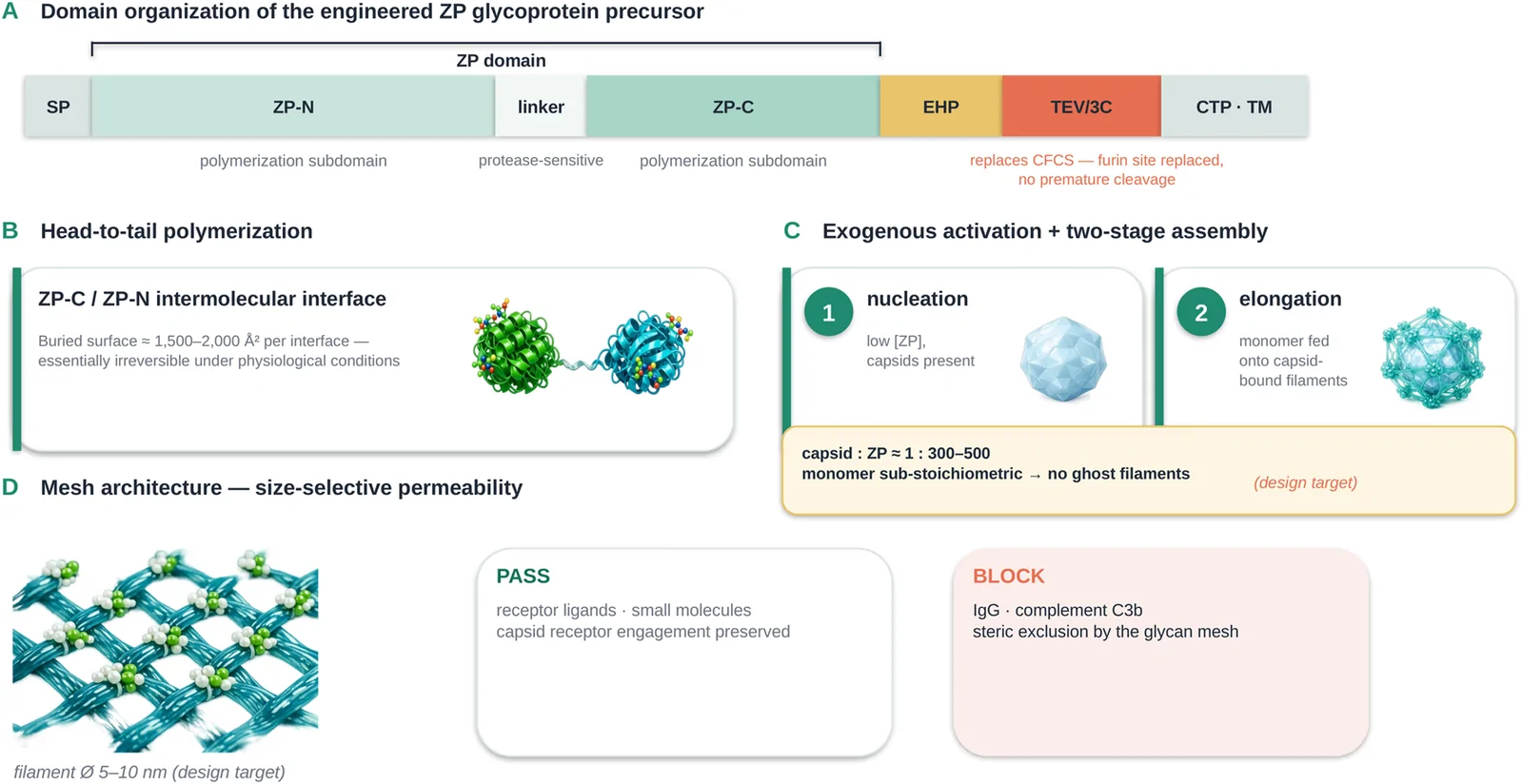
Molecular mechanism of ZP domain polymerization and capsid coating. **(A)** Domain organization of the ZP glycoprotein precursor: ZP-N subdomain, protease-sensitive linker, ZP-C subdomain, and C-terminal propeptide (CTP) containing the external hydrophobic patch (EHP) and the consensus furin cleavage site (CFCS). **(B)** Head-to-tail polymerization via ZP-C/ZP-N intermolecular interactions (buried surface area ∼1,500–2,000 Å^2^ per interface). **(C)** Exogenous protease-controlled activation strategy: replacement of the CFCS with tobacco, etch virus (TEV) or human rhinovirus 3C protease recognition sequences prevents premature polymerization during manufacturing; coating is initiated on demand by exogenous protease addition. **(D)** Mesh architecture with 5–10 nm filaments and defined inter-filament pores permitting receptor accessibility while blocking antibody and complement penetration.

### 2.5 Computational modeling of coating nucleation and AND-Gate kinetics

To move the framework beyond qualitative reasoning, we formulated two minimal analytical models. First, a nucleation-and-growth model of coating formation treats capsid-surface assembly as heterogeneous nucleation: the lag time scales as t_lag ≈1/(k_n × N_s), where k_n is the effective nucleation rate constant and N_s the number of capsid-surface nucleation sites, and subsequent growth is first-order in free monomer until depletion. With k_n estimated from nucleation-dependent protein-assembly systems of comparable interface size and N_s ≈ 50–200 affinity tags per capsid, the model predicts lag phases of ≈ 5–15 min and coating completion within 30–60 min at 10–50 μM ZP, internally consistent with the parameters given in Section 2.3; critically, it predicts that bulk (homogeneous) nucleation remains negligible provided the free-monomer concentration stays below the critical aggregation concentration throughout activation. Second, a sequential kinetic model of the AND-gate describes payload availability through three compartment-coupled first-order steps with rate constants k_MMP (tumor protease remodeling), k_pH (endosomal escape) and k_GSH (cytosolic disassembly). In target tissue (all three k_i > 0) the model predicts near-complete nuclear delivery with a characteristic sigmoidal delay, whereas in off-target tissue (k_MMP ≈0) predicted payload release remains <5% of the input dose over a 48 h horizon, compared with 40%–70% for an otherwise identical single-stimulus design. Both models are deliberately parsimonious: their role is to define the quantitative expectations that an experimental campaign must reproduce or refute (Section 5.5), and their parameters are stated as literature-derived priors rather than fitted values. Molecular dynamics simulation of the ZP-N/ZP-C–capsid interface and coarse-grained modeling of mesh assembly constitute the natural next tier of computational validation.

## 3 Triple-stimuli responsive AND-Gate release

### 3.1 Protease-sensitive linkers for tumor microenvironment activation

The triple-stimuli responsive architecture was designed to enable sequential activation logic wherein complete payload release requires progression through multiple intracellular compartments. This AND-gate behavior addresses fundamental concerns about temporal incompatibility of individual triggers and ensures therapeutic payload access to nuclear import machinery occurs only after successful transit through both membrane barriers (Mura et al., 2013). The design of each responsive element was guided by established principles from controlled release literature, adapted to the specific context of intracellular gene delivery.

Protease-cleavable linkers were incorporated for triggered ZP layer remodeling in disease-specific microenvironments. The tumor microenvironment is characterized by elevated expression of matrix metalloproteinases (MMPs), particularly MMP-2 and MMP-9, secreted by tumor cells, cancer-associated fibroblasts, and tumor-associated macrophages to degrade extracellular matrix components and facilitate invasion and metastasis (Guo et al., 2024; Zhang et al., 2024). By incorporating peptide substrates validated for MMP-mediated cleavage in tumor-targeting delivery systems, the ZP-SVV coating can be specifically remodeled at tumor sites. This remodeling exposes hidden targeting ligands previously shielded within the coating, effectively converting vectors from a stealth to a targeting-competent state exclusively in diseased tissue (Son et al., 2023).

Multi-protease responsive systems requiring combinatorial cleavage enhance specificity for protease-co-enriched tumor environments while minimizing activation in healthy tissues expressing only single proteases at low levels. The logical AND-gate design ensures coating remodeling and payload exposure occur only when the complete protease signature of the target tissue is encountered (Zong et al., 2024). For example, linkers requiring sequential cleavage by both MMP-2 and cathepsin B would only be fully processed in tumor microenvironments where both proteases are elevated, whereas healthy tissues expressing basal MMP-2 would not trigger complete remodeling. This combinatorial specificity represents a significant advance over conventional single-protease responsive systems.

### 3.2 pH-gated endosomal escape mechanism

pH-sensitive conformational switches were engineered for endosomal escape. Histidine-rich sequences undergo protonation at acidic endosomal pH, introducing positive charges that disrupt electrostatic interactions within the ZP layer and mediate membrane insertion through amphipathic helix formation (Jain et al., 2025). The pH-dependent protonation of histidine residues, with pK_a_ approximately 6.0–6.5 in polypeptide contexts, enables a sharp charge transition precisely within the endosomal pH window. At extracellular pH 7.4, histidine residues remain largely unprotonated and neutral, maintaining coating integrity. Upon endosomal acidification to pH 6.0 in early endosomes and pH 4.5 in lysosomes, progressive protonation introduces increasing positive charge density (Merkel et al., 2009).

Recent advances demonstrate enhanced transfection efficiency using histidylated peptides: histidine-substituted cell-penetrating peptides exhibit pH-dependent membrane-perturbation activity enabling efficient cytosolic delivery of functional proteins (Pack et al., 2005). The proton sponge effect of histidine-rich sequences contributes to endosomal swelling and rupture through chloride ion influx and osmotic pressure increase, mechanisms well-documented for polyhistidine-containing delivery vehicles (Putnam, 2006). The number and spacing of histidine residues can be tuned to modulate pH threshold and cooperativity, enabling optimization for specific endosomal trafficking kinetics (Dobrovolskaia and McNeil, 2007).

### 3.3 Redox-responsive disulfide crosslinks

Redox-responsive disulfide crosslinks were engineered for intracellular payload release. Eight conserved cysteines in the ZP domain participate in structural disulfide bonds contributing to protein stability and filament integrity. Additional cysteine residues introduced at strategic positions create reducible cross-links stable in the oxidizing extracellular environment but rapidly cleaved by high glutathione concentrations in the cytosol (Meng et al., 2009). The extracellular environment maintains an oxidizing redox potential favoring disulfide stability, whereas the cytosol provides a reducing environment with glutathione concentrations of 2–10 mM compared to approximately 2 μM extracellularly, creating a sharp redox gradient across cellular membranes (Cheng et al., 2011).

Reduction-sensitive polymers containing disulfide bonds undergo rapid cleavage in intracellular reducing environments while remaining stable in circulation (Sahay et al., 2013). The second-order kinetics of disulfide reduction by glutathione have been characterized in various bioconjugate systems. Multi-triggered release coordinates pH-triggered endosomal escape with glutathione-mediated cytosolic disassembly, ensuring payload access to nuclear import machinery occurs only after successful transit through both membrane barriers (Wittrup and Lieberman, 2015). This coordination prevents premature cytosolic release of payloads that fail to escape endosomes, and is predicted to improve nuclear delivery efficiency relative to single-trigger systems.

### 3.4 Sequential AND-Gate logic and release specificity

The temporal sequence of AND-gate activation proceeds through three strictly ordered steps. Step 1 (Extracellular): Tumor-associated protease cleavage exposes cryptic targeting ligands and initiates coating loosening without payload release. Step 2 (Endosomal): Receptor-mediated endocytosis delivers vectors to early endosomes, where histidine protonation triggers membrane insertion and endosomal escape; covalent disulfide crosslinks maintain structural integrity, preventing premature uncoating. Step 3 (Cytosolic): Intracellular glutathione reduces engineered disulfides, completing coating disassembly and releasing the viral core for nuclear trafficking. The design specifications, anatomical sites, and functional outcomes of the three responsive modules are summarized in Table 1. This hierarchical logic ensures that off-target tissues lacking the complete trigger set cannot activate premature payload release (Sago et al., 2018).

**TABLE 1:** Design specifications of the triple-stimuli responsive modules of the ZP-SVV platform.

Module	Anatomical site	Environmental trigger	Engineered molecular element	Functional outcome	Key references
Protease-responsive remodeling	Tumor microenvironment (extracellular)	MMP-2/9 co-elevation (with optional cathepsin B gating)	Peptide substrate linkers inserted into the ZP coating	Stealth-to-targeting switch; exposure of cryptic ligands; coating loosening	(Guo et al., 2024; Zhang et al., 2024; Son et al., 2023; Zong et al., 2024)
pH-gated escape	Early/late endosome	Endosomal acidification (pH 6.0 → 4.5)	Histidine-rich domains (pK_a_ 6.0–6.5)	Proton-sponge-mediated endosomal escape with coating integrity preserved	(Jain et al., 2025; Merkel et al., 2009; Pack et al., 2005; Putnam, 2006; Dobrovolskaia and McNeil, 2007)
Redox-responsive disassembly	Cytosol	Glutathione (2–10 mM)	Engineered disulfide crosslinks	Complete coating disassembly; release of the viral core for nuclear trafficking	(Meng et al., 2009; Cheng et al., 2011; Sahay et al., 2013; Wittrup and Lieberman, 2015)

MMP, matrix metalloproteinase.

Tissues deficient in tumor-specific proteases cannot initiate the cascade, even in the presence of basal endosomal acidification and cytosolic glutathione. Similarly, vectors that escape the tumor microenvironment without protease encounter remain in a stealth state and are cleared without payload release. This multi-layered filtering is predicted to markedly reduce off-target toxicity compared with single-stimuli systems (Ganta et al., 2008) (Section 2.5). The kinetic advantage of sequential logic lies in its stepwise filtration: protease cleavage provides tissue selectivity, pH response ensures endosomal escape competence, and redox sensitivity guarantees cytosolic localization prior to payload release. The sequential activation cascade, theoretical release profiles, intracellular trafficking trajectory, and combinatorial trigger requirement are illustrated in Figure 3.

**FIGURE 3:**
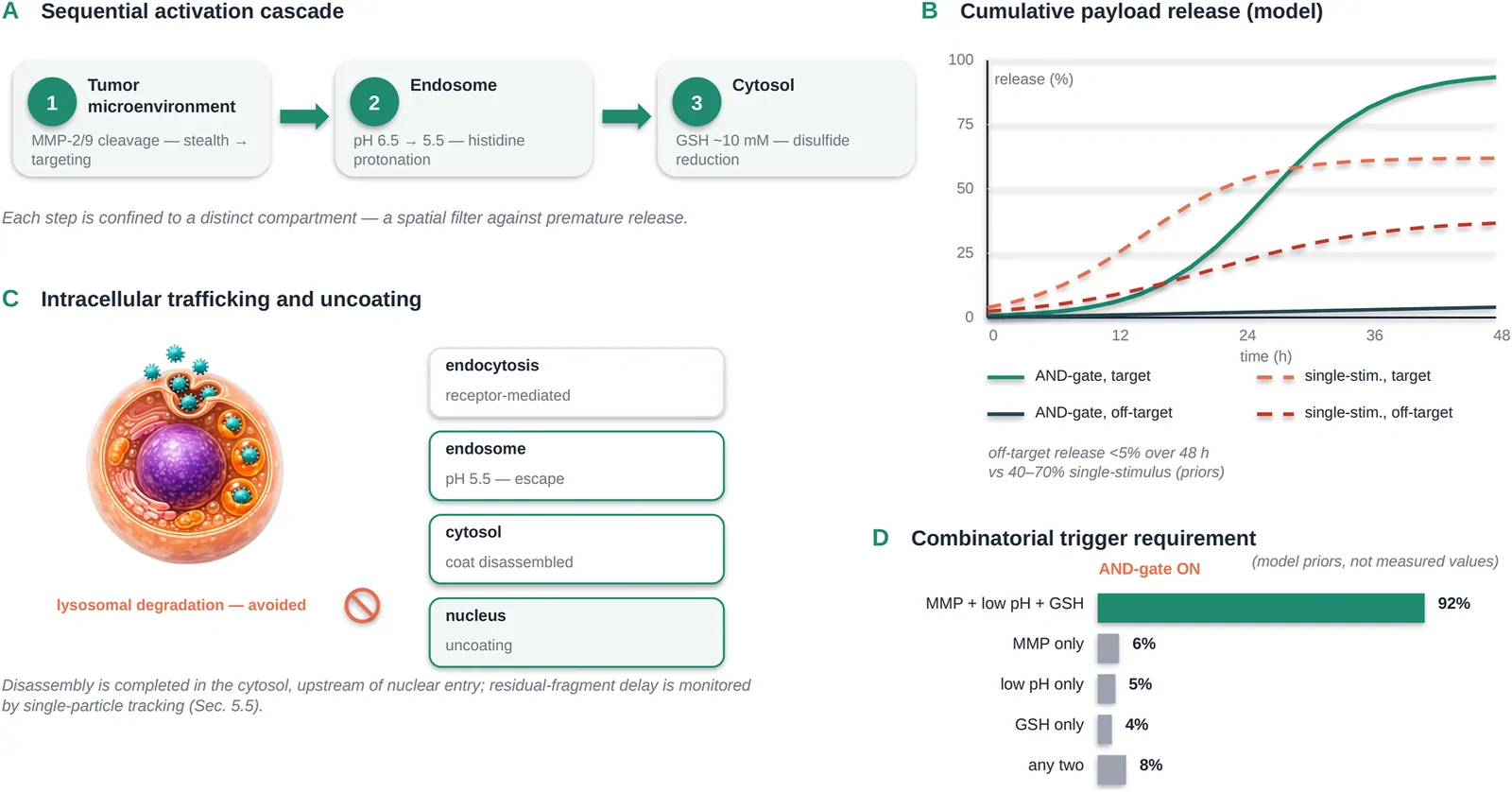
Triple-stimuli responsive AND-gate release kinetics and spatiotemporal precision. **(A)** Sequential activation cascade: matrix metalloproteinase (MMP-2/9) cleavage in the tumor microenvironment (Step 1) → histidine protonation during endosomal acidification (Step 2) → glutathione (GSH)-mediated disulfide reduction in the cytosol (Step 3). **(B)** Theoretical cumulative payload release profiles comparing the AND-gate system in target versus off-target tissues (solid lines) with single-stimuli systems (dashed lines), demonstrating minimal premature release in off-target tissues. **(C)** Intracellular trafficking trajectory showing receptor-mediated endocytosis, pH-gated endosomal escape, and avoidance of lysosomal degradation. **(D)** Combinatorial trigger requirement: theoretical payload release occurs only when the complete MMP + low pH + GSH trigger set is present. Values shown in **(B,D)** are theoretical predictions.

Compatibility with native AAV trafficking and uncoating. The coat must also leave the capsid’s intracellular program intact—externalization of the VP1 N-terminal phospholipase A2 domain during endosomal acidification, endosomal escape, nuclear import, and uncoating. Docking is non-covalent, through capsid-surface affinity tags, and the inter-filament pores (tens of nm) are far larger than the small-amplitude displacement required for VP1 externalization; Step-2 pH-gated loosening enlarges local porosity further within the endosome. Disassembly is completed in the cytosol (Step 3), upstream of nuclear entry, so the viral core reaches the nuclear pore unencumbered—the engineered disulfides that hold the mesh together are precisely the elements cleaved at that stage. Because AAV uncoating occurs at or within the nucleus (Domenge et al., 2019), genome release is unimpeded once the core arrives. Residual coat fragments could in principle delay endosomal escape; single-particle live-cell tracking of coated versus uncoated vectors is therefore included in the validation agenda (Section 5.5).

## 4 Multi-modal immune evasion

### 4.1 Dense glycan shielding

The immune evasion strategy of ZP-SVV integrates multiple complementary mechanisms rather than relying on single approaches. This multi-layered design provides redundancy addressing diverse recognition mechanisms employed by the immune system against foreign vectors (Dassie et al., 2009). The effectiveness of immune evasion depends on simultaneously addressing humoral immunity, complement activation, and cellular phagocytosis through distinct but complementary molecular mechanisms.

The dense glycan shield masks viral epitopes through combined steric barrier and self-like presentation, analogous to the glycan shields of HIV-1 and SARS-CoV-2 (Zhong et al., 2024). Its effectiveness rests on the density, structure, and dynamics of the glycan layer. High-density glycosylation creates a physical barrier exceeding the reach of antibody paratopes (≈5 nm), and dense layers with oligosaccharide chains extending 10–15 nm effectively prevent binding by steric exclusion (Li et al., 2023; Doores, 2015); the multiple N-linked glycosylation sites on each ZP domain make such densities theoretically attainable. Complex-type sialylated N-glycans are the preferred structure because they mimic the host glycocalyx, whereas high-mannose glycans are typically read as foreign (Joyce et al., 2015). The rigidity of ZP protein β-strands, finally, holds glycans in extended conformations and provides consistent coverage through the inter-filament pores, whereas flexible PEG chains can collapse and expose the underlying surface (Gabrilovich and Nagaraj, 2009).

### 4.2 Complement system evasion

Complement system evasion is achieved through multiple mechanisms collectively preventing membrane attack complex (MAC) formation. Sialic acid-terminated glycans engage CD59 and other membrane-bound regulatory proteins inhibiting complement activation at the C8/C9 stages (Ricklin et al., 2010). The coating recruits endogenous complement regulators from body fluids including factor H and C4-binding protein. Additionally, steric hindrance of large complement component deposition while permitting smaller molecule access creates selective barriers preventing initiation of the classical and alternative complement pathways (Kropf et al., 2024). Recent evidence highlights complement activation as a major barrier to systemic AAV administration, with clinical trials demonstrating thrombotic microangiopathy linked to complement activation at high vector doses (Salabarria et al., 2024; Benemei et al., 2024).

Zaiss et al. (2008) demonstrated that complement is an essential component of the immune response to AAV vectors, with capsid-specific antibodies triggering activation primarily through the classical pathway, alongside alternative pathway contributions. The multi-layered complement evasion strategy addresses this through active recruitment of regulatory proteins and passive steric blockade. Factor H recruitment is particularly important as it regulates alternative pathway amplification loops that can rapidly generate complement deposition on foreign surfaces (Ricklin et al., 2010). By presenting sialic acid residues and sulfated glycans similar to the host glycocalyx, the ZP coating effectively hijacks natural complement regulatory mechanisms.

### 4.3 Macrophage phagocytosis resistance via siglec engagement

Macrophage phagocytosis resistance is achieved through alternative inhibitory pathways despite the absence of CD47. Sialic acid-containing glycans engage inhibitory siglec receptors on macrophages and related phagocytic cells (Saini et al., 2022). Many siglecs contain immunoreceptor tyrosine-based inhibition motifs (ITIMs) that suppress phagocytic signaling upon ligand engagement. Engagement of Siglec-10 or related inhibitory siglecs by sialylated glycans triggers inhibitory signaling cascades counteracting pro-phagocytic signals, effectively creating phagocytosis-inhibitory signals analogous to CD47–SIRP-α interactions but achieved through glycan-mediated recognition (Barkal et al., 2019).

Recent studies highlight the Siglec–sialic acid axis as a novel immune checkpoint in cancer. Daly et al. (2019) reviewed how inhibitory siglecs on immune cells modulate anti-tumor immunity when engaged by sialylated glycans. Wang J. et al. (2019) identified Siglec-15 as an immune suppressor and a potential target for normalization cancer immunotherapy, while Mei et al. (2023) showed that Siglec-9 acts as an immune-checkpoint molecule on glioblastoma-associated macrophages, restricting T-cell priming and immunotherapy response. Gonzalez-Gil and Schnaar (2021) have comprehensively characterized Siglec ligands on immune cells, including sialylated glycan structures that regulate T-cell and natural killer cell activation. By presenting appropriate sialylated glycan patterns, ZP-SVV engages these inhibitory pathways to evade phagocytic clearance.

### 4.4 ECM mimicry and theoretical predictions of immune evasion efficiency

ECM mimicry provides additional immune evasion by presenting familiar molecular patterns that fail to trigger pattern recognition receptor signaling. The ZP glycoprotein matrix naturally resembles extracellular matrix components, reducing activation of dendritic cells and antigen-presenting cells (McNally, 2024). The glycan components of ZP proteins, including sulfated glycans and hyaluronic acid-like motifs, mimic natural ECM compositions recognized as self by the innate immune system. Combined with glycan shielding masking foreign protein epitopes and siglec engagement actively suppressing phagocytosis, this multi-mechanism approach achieves comprehensive immune evasion comparable to or exceeding CD47-based strategies, without requiring membrane extraction and fusion procedures (Laforet, 2025). The redundancy ensures that even if one evasion mechanism is partially compromised, others maintain protection.

Theoretical considerations suggest that the multi-modal immune evasion approach could provide broader protection than single-mechanism strategies; the estimates below are order-of-magnitude priors derived from published heterologous systems, not fitted values. Based on our quantitative analysis of published binding constants and cellular response thresholds, we predict that the glycan shield would reduce antibody binding affinity by 10- to 100-fold through steric exclusion alone. Similarly, we estimate that siglec engagement on macrophages would reduce phagocytic uptake by 50%–80% based on reported ITIM signaling strengths, and that complement evasion through factor H recruitment would reduce C3b deposition by 60%–90% depending on sialic acid density (Ricklin et al., 2010). The combined effect follows multiplicative rather than additive kinetics, as each mechanism targets distinct recognition pathways. Compared to RBCM coating, which relies primarily on CD47–SIRP-α signaling, the ZP-SVV approach provides mechanistic diversity that may prove more robust across diverse patient populations and disease contexts (Saini et al., 2022). Each estimate corresponds to an explicit, falsifiable prediction (Section 5.5, P2–P4). The predicted magnitudes of each evasion mechanism and their multiplicative combination are summarized in Figure 4.

**FIGURE 4:**
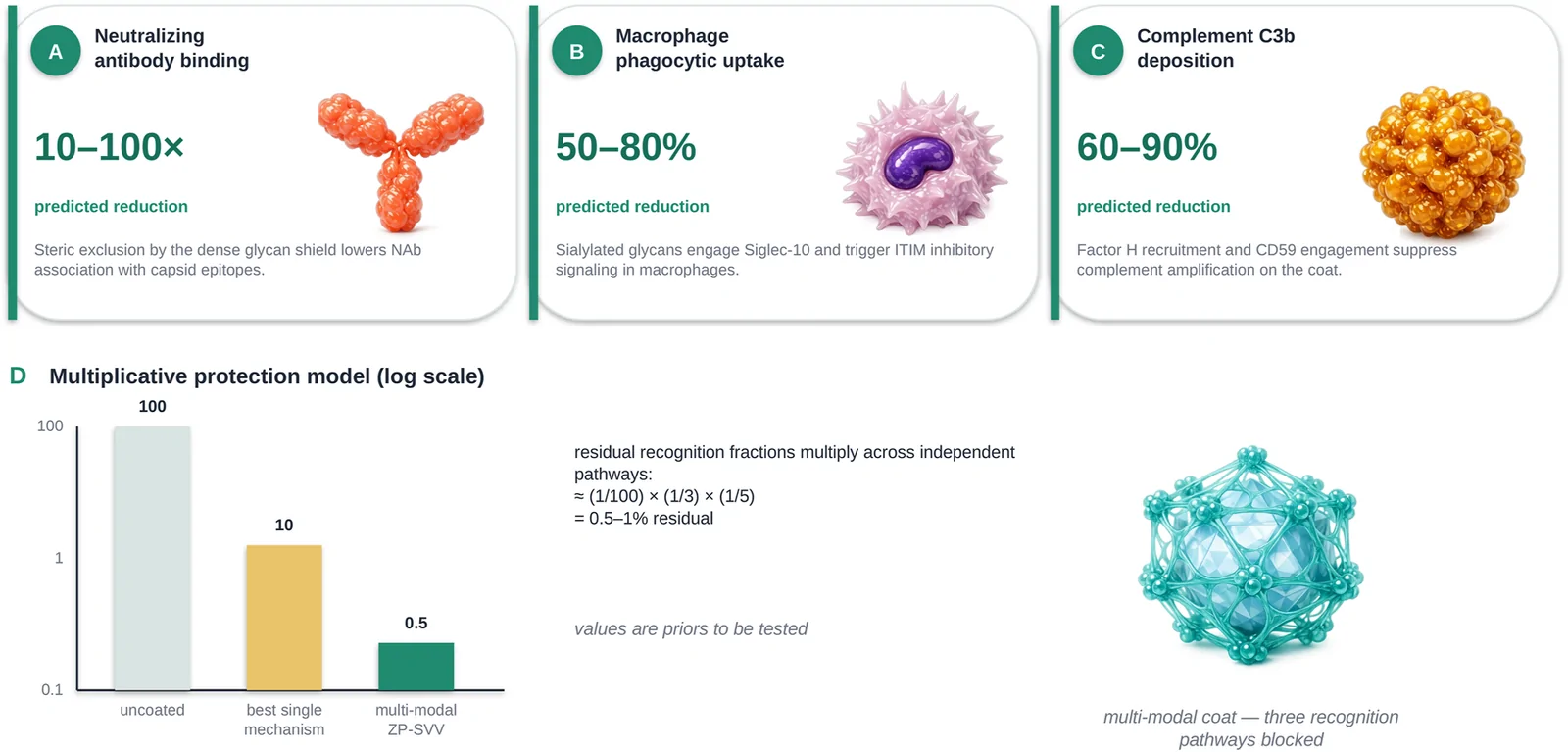
Theoretical prediction of multi-modal immune evasion efficiency. **(A)** Predicted neutralizing antibody (NAb) binding relative to uncoated AAV, showing a 10- to 100-fold reduction via glycan shield steric exclusion. **(B)** Predicted macrophage phagocytic uptake, showing a 50%–80% reduction through Siglec-10 engagement by sialylated glycans. **(C)** Predicted complement C3b deposition, showing a 60%–90% reduction via factor H recruitment and CD59 engagement. **(D)** Multiplicative protection model comparing combined multi-modal evasion with the best single-mechanism strategy. All values are theoretical predictions derived from published binding constants and cellular response thresholds. All ranges are order-of-magnitude priors intended as testable predictions (Section 5.5).

## 5 Discussion

The ZP-SVV design integrates three functional layers—self-assembling coating, AND-gate responsiveness, and multi-modal immune evasion—into a single genetically encoded platform. The following discussion positions this design against existing biomimetic strategies, outlines its most compelling therapeutic applications, and critically examines the limitations that must be addressed before clinical translation.

### 5.1 Comparison with existing biomimetic platforms

Red blood cell membrane-wrapped AAV represents the most clinically advanced biomimetic platform, exploiting CD47-mediated phagocytosis inhibition and complete viral epitope masking (Rodriguez et al., 2013). However, RBCM limitations are addressable by ZP-SVV: passive stealth without intrinsic active targeting capability, membrane fragility against shear forces and serum lipoprotein interactions, and complete surface coverage interfering with vector–cell interactions by masking capsid receptor-binding sites (Hu et al., 2015). ZP glycoprotein matrices may offer complementary advantages: greater mechanical stability via covalent cross-linking, preserved receptor accessibility through mesh-like architecture with inter-filament pores, and integrated targeting and responsive functionality through domain engineering not requiring additional manufacturing steps (Hu et al., 2011).

Exosome-based delivery offers inherent biocompatibility and natural targeting but faces scalability, consistency, and loading efficiency challenges limiting clinical translation (Pegtel and Gould, 2019). PEGylation achieves immune evasion through steric exclusion but at the cost of reduced cellular uptake and receptor accessibility. The flexible unstructured nature of PEG chains creates hydrated layers preventing both immune recognition and receptor binding (Harris and Chess, 2003). In contrast, ZP-SVV glycan shield provides structured rigid glycan presentation maintaining steric exclusion of immune components while permitting receptor access through defined inter-filament pores. This structural distinction raises the possibility that ZP-SVV could achieve effective immune evasion without the cellular uptake penalties associated with PEGylation (Weinbaum et al., 2007), a prediction that requires experimental verification (Section 5.5).

To render this comparison systematic rather than descriptive, Table 2 summarizes the four platforms across the dimensions most relevant to clinical translation.

**TABLE 2:** Systematic comparison of ZP-SVV with existing biomimetic stealthing strategies.

Dimension	ZP-SVV	PEGylation	RBC membrane coating	Exosome-based delivery
Primary immune evasion mechanism	Structured glycan shield + complement regulation + siglec engagement (multi-modal)	Steric hydration layer	CD47–SIRP-α signaling + epitope masking	Endogenous self-vesicle identity
Humoral/complement evasion	Designed (factor H recruitment, CD59 engagement)	Partial; anti-PEG immunity reported	Partial	Partial
Targeting capability	Cryptic ligands unveiled by protease gating	Passive only	Limited (membrane proteins)	Natural tropism, variable
Stimuli responsiveness	Triple AND-gate (protease/pH/redox)	None	None	Limited
Receptor accessibility	Preserved via inter-filament pores	Masked by PEG corona	Masked by membrane	Preserved
Manufacturing complexity	Genetically encoded self-assembly; glycoform control required	Chemical conjugation; heterogeneous sites	Membrane extraction and fusion; batch variability	Isolation and loading; scale-up difficult
Scalability and consistency	Potentially high (defined components)	High	Low–moderate	Low
Key clinical translation challenges	ZP immunogenicity; glycoform homogeneity	Anti-PEG antibodies; uptake penalty	Membrane stability; source supply	Yield; loading capacity; heterogeneity

### 5.2 Therapeutic applications in reproductive medicine and oncology

AAV1 and AAV-DJ exhibit intrinsic ZP penetration capability, enabling oocyte access without invasive micromanipulation for reproductive medicine applications. ZP-SVV enhances this natural capability through ZP glycoprotein-derived sequences mediating specific binding and transport across the zona pellucida barrier (Evans, 2012). Clinical indications include maternal-effect mutation correction in genes including NLRP7, KHDC3L, PADI6, TUBB8, PATL2, and WEE2, which cause preimplantation embryonic lethality or severe developmental disorders (Nagaoka et al., 2012). Conventional preimplantation genetic testing identifies affected embryos but cannot correct underlying mutations. ZP-SVV-mediated gene delivery to oocytes offers an alternative strategy for correcting mutations before embryonic genome activation.

For oncology applications, the protease-rich tumor microenvironment provides ideal activation triggers, with MMP-2 and MMP-9 levels frequently elevated in solid tumors due to the activity of tumor-associated macrophages, cancer-associated fibroblasts, and tumor cells (Wickham et al., 1997; Arap et al., 1998). Multi-protease responsive systems enhance specificity, while immune evasion enables sustained circulation and tumor accumulation of vectors encoding checkpoint inhibitors, cytokines, or immunomodulators (Jain, 2012). The controlled release kinetics in tumor microenvironments can be modeled as sequential reaction cascades: initial protease-triggered remodeling exposes targeting ligands, subsequent endosomal escape and glutathione-mediated uncoating release therapeutic payloads specifically within tumor cells. This staged control is designed to minimize systemic exposure while concentrating payload within tumors, two long-standing limitations of conventional systemic gene therapy. Figure 5 illustrates both application scenarios and provides a comparative assessment of the ZP-SVV platform against existing biomimetic delivery strategies.

**FIGURE 5:**
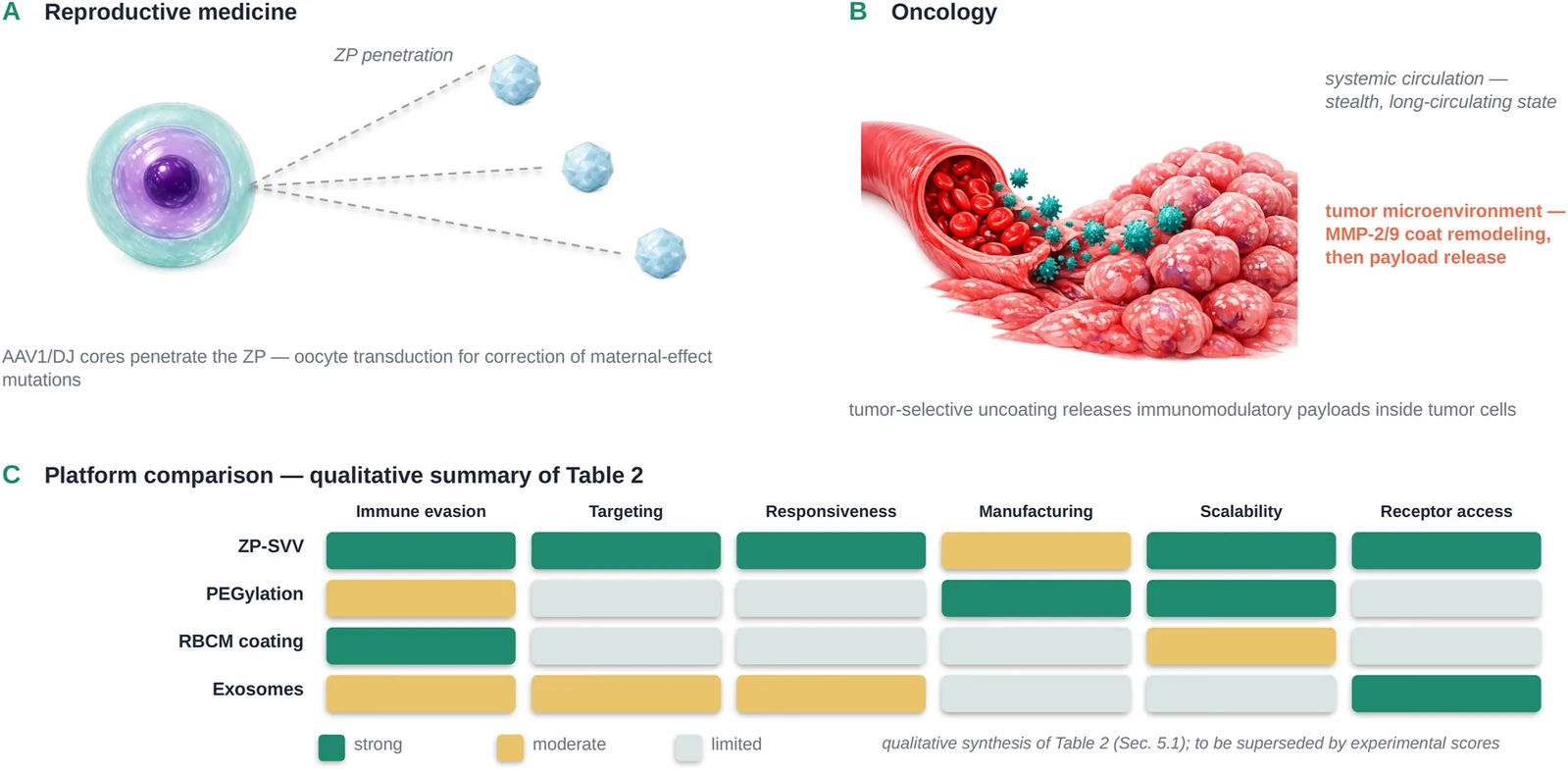
Therapeutic applications and platform comparison. **(A)** Reproductive medicine: ZP-SVV-mediated oocyte gene delivery leveraging intrinsic AAV1/DJ zona pellucida penetration for maternal-effect mutation correction. **(B)** Oncology: tumor microenvironment-activated remodeling and controlled release of immunomodulatory payloads. **(C)** Radar chart comparing ZP-SVV, PEGylation, red blood cell membrane (RBCM) coating, and exosome-based delivery across six performance metrics: immune evasion, targeting precision, manufacturing scalability, receptor accessibility, stimuli responsiveness, and mechanical stability. Scores (1–5) represent a qualitative theoretical assessment based on the comparative analysis in Section 5.1.

### 5.3 Limitations and translational considerations

Several limitations of the present theoretical framework must be acknowledged. First, the ZP-SVV platform is a rational design supported by evolutionary analogy and theoretical modeling; none of the predicted performance parameters—including the 10- to 100-fold reduction in antibody binding, the 50%–80% reduction in phagocytic uptake, and the AND-gate release fidelity—has been validated experimentally. The quantitative estimates derive from published binding constants and cellular response thresholds obtained in heterologous systems, and their transferability to ZP-coated AAV particles requires direct experimental testing.

Second, the immunogenicity of ZP glycoproteins themselves warrants careful evaluation. Although the glycan shield is designed to present self-like molecular patterns, ZP glycoproteins are not ubiquitously expressed in adults, and anti-ZP antibodies have been described in the reproductive biology literature; whether engineered ZP coatings could elicit antibody or T-cell responses upon repeated systemic administration remains unknown and must be characterized in relevant animal models before clinical translation.

The reproductive biology literature provides a direct cautionary precedent. ZP glycoproteins have long been investigated as contraceptive-vaccine antigens precisely because they can be immunogenic in non-self contexts: immunization with ZP3 or its immunodominant T-cell epitopes induces high-titer anti-ZP antibodies and, in some mouse models, T-cell-mediated autoimmune oophoritis and ovarian failure (Rhim et al., 1992); conversely, carefully engineered ZP3 peptide vaccines can elicit contraceptive antibodies without ovarian pathology, demonstrating that epitope choice determines the safety outcome (Lou et al., 1995). A systemically administered ZP-SVV must therefore be engineered to fall on the safe side of this precedent, particularly in female patients of reproductive age.

Four mitigation layers follow from this precedent. The coating uses human ZP sequences—autologous self-proteins for any recipient—so central tolerance should cover most ZP-derived epitopes, and the residual risk concentrates in epitopes not presented during thymic education. Where that margin proves insufficient, computational T-cell epitope mapping will guide conservative point substitutions (deimmunization), avoiding in particular the immunodominant ZP3 epitopes characterized in the contraceptive-vaccine literature (Rhim et al., 1992; Lou et al., 1995); ZP1/ZP2-based coats provide a fallback where epitope removal is incompatible with assembly. Exposure is limited by design, since the coat disassembles upon cell entry, restricting antigen persistence, and most gene therapy indications involve single-course administration. Finally, the validation agenda includes longitudinal monitoring of anti-ZP IgG/IgM titers together with ovarian histopathology in repeat-dose rodent studies as a pre-specified safety endpoint (Section 5.5).

It is also reasonable to ask why ZP should be preferred over other human-derived extracellular matrix components (laminin, fibronectin, collagen fragments) or endogenous camouflage strategies such as CD47 display. ZP glycoproteins uniquely combine, within a single genetically encoded module, spontaneous covalently stabilized polymerization into a mesh of defined porosity, dense and tunable glycosylation, and natural competence for protease-mediated remodeling. Integrin-binding ECM proteins would introduce unwanted signaling through cell-surface integrins, and CD47-based camouflage addresses phagocytosis but neither humoral immunity nor controlled release. We therefore regard ZP as the only human-derived scaffold that presently combines all three required functions, while acknowledging that its reproductive immunology history mandates the layered mitigations described above.

Third, manufacturing challenges are non-trivial. Glycosylation homogeneity is critical for both immune evasion and complement regulation, yet scalable production of defined glycoforms remains technically demanding despite advances in glycoengineered cell lines and *in vitro* glycan remodeling (Meuris et al., 2014). The exogenous protease activation step introduces an additional unit operation requiring tight process control and clearance validation.

Finally, the theoretical framework does not yet account for inter-patient variability in protease expression, endosomal pH dynamics, or cytosolic redox states, all of which may influence AND-gate fidelity in clinical settings. These limitations define a clear experimental agenda: *in vitro* assembly and biophysical characterization of ZP-coated capsids, quantitative assessment of immune evasion in NAb-positive sera, and *in vivo* evaluation of targeting specificity, biodistribution, and safety. We therefore present the ZP-SVV concept as a testable design hypothesis intended to stimulate such experimental efforts.

### 5.4 Established principles versus proposed hypotheses

To avoid conflating validated biology with engineering proposals, Table 3 classifies the platform’s constituent elements by evidentiary status. Elements marked as proposed should be read as hypotheses to be tested, not as established performance characteristics.

**TABLE 3:** Evidentiary status of the platform’s constituent elements.

Element	Status	Evidence base
ZP domain head-to-tail polymerization (ZP-N/ZP-C)	Established	Structural and *in vitro* assembly studies (Monné and Jovine, 2011; Han et al., 2010; Jovine et al., 2002; Wassarman, 2008; Raj et al., 2017; Litscher and Wassarman, 2020; Bokhove et al., 2016; Schaeffer et al., 2009)
Proteolytic activation of ZP precursors (CFCS/furin)	Established	Reproductive biology literature (Kiefer and Saling, 2002; Thomas, 2002)
Glycan-shield immune evasion by steric exclusion	Established (viral contexts)	HIV/SARS-CoV-2 glycan shield literature (Li et al., 2023; Zhong et al., 2024)
Siglec–sialic acid inhibitory signaling	Established	(Saini et al., 2022; Barkal et al., 2019; Daly et al., 2019; Wang J et al., 2019; Mei et al., 2023; Gonzalez-Gil and Schnaar, 2021)
Complement regulation by sialic acid/factor H	Established	(Ricklin et al., 2010; Kropf et al., 2024; Salabarria et al., 2024; Benemei et al., 2024; Zaiss et al., 2008)
MMP-, pH- and GSH-responsive elements (individually)	Established (non-viral carriers)	(Guo et al., 2024; Zhang et al., 2024; Son et al., 2023; Zong et al., 2024; Jain et al., 2025; Merkel et al., 2009; Pack et al., 2005; Putnam, 2006; Dobrovolskaia and McNeil, 2007; Meng et al., 2009; Cheng et al., 2011; Sahay et al., 2013; Wittrup and Lieberman, 2015)
ZP glycoprotein coating on AAV capsids	Proposed	This work
Surface-selective, ghost-filament-free assembly	Proposed	This work (Sections 2.2, 2.4, 2.5)
Triple AND-gate gating on a virion	Proposed	This work (Section 3)
Quantitative performance estimates (Figure 4)	Proposed (order-of-magnitude priors)	Extrapolated from (Li et al., 2023; Ricklin et al., 2010; Kropf et al., 2024; Salabarria et al., 2024; Benemei et al., 2024; Zaiss et al., 2008; Saini et al., 2022; Barkal et al., 2019; Daly et al., 2019; Wang J et al., 2019; Mei et al., 2023; Gonzalez-Gil and Schnaar, 2021)

### 5.5 Testable predictions and falsification criteria

We define the following predictions, each with an explicit falsification criterion and a corresponding assay (summarized in Figure 6). Failure of any single prediction does not invalidate the platform as a whole, but identifies which module requires redesign.

**FIGURE 6:**
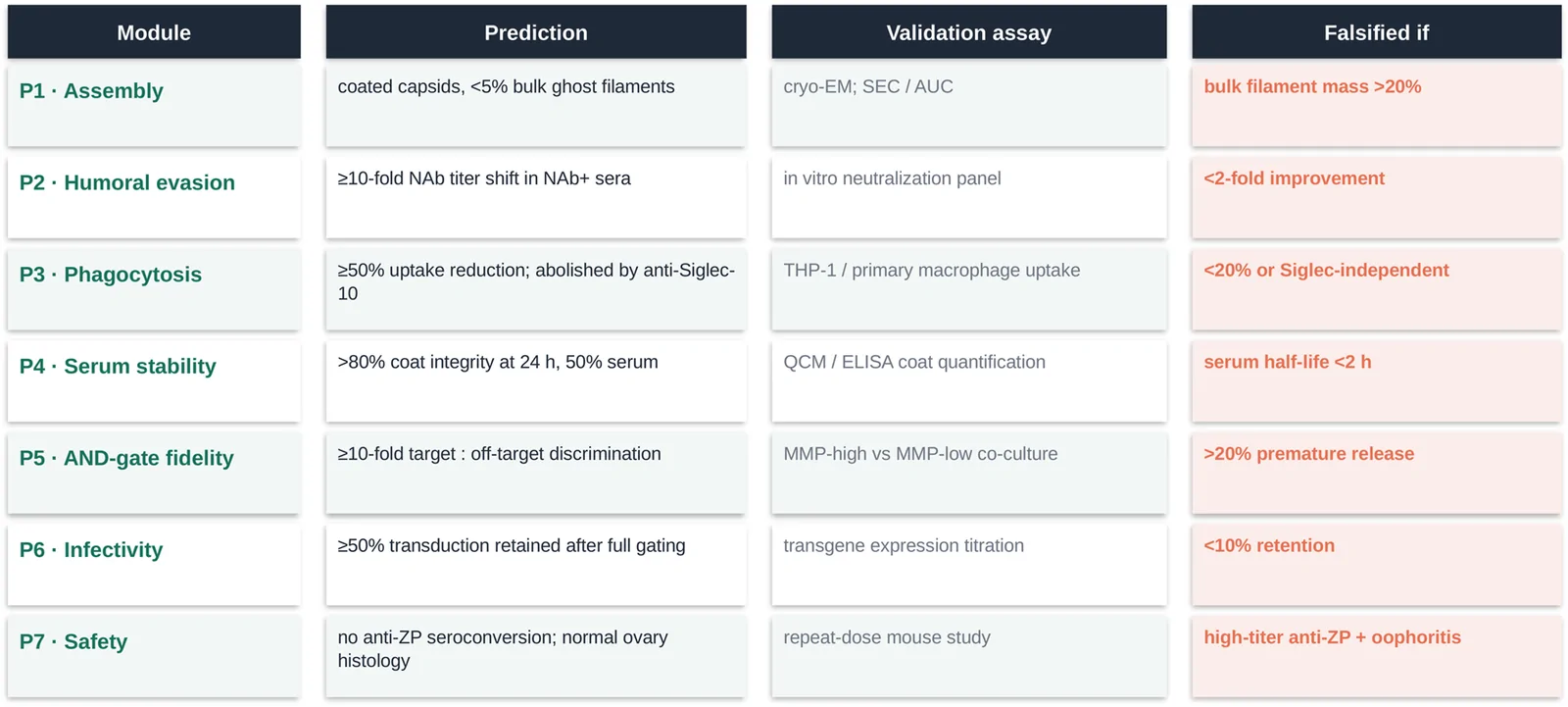
Testable predictions and falsification criteria of the ZP-SVV platform. For each module (P1–P7), the predicted outcome, the corresponding validation assay, and the pre-specified falsification threshold are shown. Failure of a single prediction identifies the module requiring redesign without invalidating the platform. All thresholds are design targets to be confirmed experimentally.

P1 — Assembly selectivity. Prediction: the optimized activation protocol yields ZP-coated capsids with <5% bulk ‘ghost’ filament mass. Assay: cryo-electron microscopy plus size-exclusion chromatography/analytical ultracentrifugation. Falsified if bulk filament mass exceeds 20% under optimized conditions.

P2 — Humoral evasion. Prediction: coating shifts the neutralization titer of NAb-positive human sera by ≥ 10-fold versus uncoated AAV. Assay: *in vitro* neutralization assay across a serum panel. Falsified if the improvement is < 2-fold.

P3 — Phagocytosis resistance. Prediction: macrophage uptake is reduced by ≥ 50% and the effect is abolished by anti-Siglec-10 blockade. Assay: THP-1 and primary macrophage uptake with and without blocking antibodies. Falsified if uptake reduction is <20% or insensitive to Siglec blockade.

P4 — Serum stability. Prediction: the coat retains >80% integrity after 24 h in 50% human serum at 37 °C. Assay: coat quantification by quartz crystal microbalance or ELISA-based filament detection. Falsified if the serum half-life is < 2 h.

P5 — AND-gate fidelity. Prediction: ≥10-fold discrimination of payload expression between MMP-high and MMP-low co-cultures. Assay: reporter transgene expression in matched co-culture models. Falsified if premature release exceeds 20% of total payload in MMP-low conditions.

P6 — Infectivity retention. Prediction: coated vectors retain ≥50% of uncoated-vector transduction in permissive cells after the complete gating sequence. Assay: transgene expression titration. Falsified if retention is <10%.

P7 — Reproductive safety. Prediction: repeat-dose administration in mice produces no sustained anti-ZP seroconversion and normal ovarian histology. Falsified if high-titer anti-ZP antibodies with oophoritis emerge; such an outcome would mandate the deimmunized, ZP3-free compositions described in Section 5.3.

## 6 Conclusion

The ZP-SVV platform repurposes the evolutionarily optimized architecture of the zona pellucida to address three persistent limitations of current AAV vectors: pre-existing immunity, poor tissue specificity, and uncontrolled intracellular release. It does so within a single, genetically encoded design that combines biomimetic self-assembly, multi-modal immune evasion, and triple-stimuli responsive release. The theoretical analysis suggests that this design may offer advantages over conventional stealthing strategies, subject to the experimental tests defined in Section 5.5.

The covalently crosslinked glycan shield is designed to provide immune evasion without compromising cellular entry, and the AND-gate architecture restricts payload release to cells presenting the complete trigger set. Because the coating self-assembles from defined components, manufacturing avoids the heterogeneity of chemical conjugation, and the modular composition allows the coat to be retuned for different indications. Reproductive medicine and oncolytic virotherapy illustrate, rather than exhaust, the settings in which such a coat could be useful.

The immediate next steps are the module-level tests specified in Section 5.5: assembly and biophysical characterization of coated capsids (P1), neutralization assays in NAb-positive sera (P2), and the repeat-dose safety study with ovarian histopathology (P7). Their outcomes will determine which modules advance to *in vivo* evaluation and which require the redesign routes already identified—deimmunized compositions, glycoengineered hosts, or alternative coat stoichiometries.

## Data Availability

The original contributions presented in the study are included in the article and its supplementary material. This is a theoretical and conceptual design study in which no new experimental data were generated; all information supporting the findings is available within the article and its references. Further inquiries can be directed to the corresponding author.
